# Protein Malnutrition Impairs Intestinal Epithelial Cell Turnover, a Potential Mechanism of Increased Cryptosporidiosis in a Murine Model

**DOI:** 10.1128/IAI.00705-16

**Published:** 2016-11-18

**Authors:** J. Liu, D. T. Bolick, G. L. Kolling, Z. Fu, R. L. Guerrant

**Affiliations:** a UVA Center for Global Health, Division of Infectious Disease and International Health, University of Virginia School of Medicine, Charlottesville, Virginia, USA; b Department of Gastrointestinal Surgery, The Third Affiliated Hospital of Sun Yat-sen University, Guangzhou, China; c Department of Pharmacology, University of Virginia School of Medicine, Charlottesville, Virginia, USA; Cornell University

## Abstract

Malnutrition and cryptosporidiosis form a vicious cycle and lead to acute and long-term growth impairment in children from developing countries. Insights into mechanisms underlying the vicious cycle will help to design rational therapies to mitigate this infection. We tested the effect of short-term protein malnutrition on Cryptosporidium parvum infection in a murine model by examining stool shedding, tissue burden, and histologic change and explored the mechanism underlying the interaction between malnutrition and cryptosporidiosis through immunostaining and immunoblotting. Protein malnutrition increased stool shedding and the number of intestine-associated C. parvum organisms, accompanied by significant suppression of C. parvum-induced caspase 3 activity and expression of PCNA and Ki67, but activation of the Akt survival pathway in intestinal epithelial cells. We find that even very brief periods of protein malnutrition may enhance (or intensify) cryptosporidiosis by suppressing C. parvum-induced cell turnover and caspase-dependent apoptosis of intestinal epithelial cells. This implicates a potential strategy to attenuate C. parvum's effects by modulating apoptosis and promoting regeneration in the intestinal epithelium.

## INTRODUCTION

Malnutrition is well recognized as a widespread health problem that can impair intestinal architecture and the host's immune system, resulting in increased vulnerability to infection (1–3). Besides its acute effects on the host, malnutrition is also considered a cause of potentially lifelong functional disability (1, 4).

Cryptosporidium is an intracellular protozoan parasite that invades the epithelial cells of the small intestine and reproduces on the apical surface of the epithelium (5). Cryptosporidiosis is now viewed as an important cause of diarrheal diseases in children and adults worldwide (6–8). In immunosuppressed patients, such as those with AIDS, cryptosporidiosis can lead to persistent diarrhea and even death (9, 10). In developing countries, persistent infections with Cryptosporidium intensified by malnutrition have been associated with impaired physical and cognitive development in children (11, 12). We have developed an easily executed model to study the interaction between protein malnutrition and C. parvum infection in weaned mice; we confirmed that in the “vicious cycle” characterized by malnutrition and C. parvum infection, each intensified the other (13, 14). However, the mechanisms underlying this vicious cycle remain unclear. Since treatment options for cryptosporidiosis are limited, insights into mechanisms responsible for the vicious circle will help to design rational therapies to mitigate this infection.

In a previous study, we demonstrated that even very brief periods (<24 h) of protein malnutrition cause rapid activation of intestinal cell kinase (ICK) as well as the Akt pathway in intestinal epithelial cells (15). In the present study, in order to determine whether these rapid intestinal cell signaling changes caused by protein malnutrition impact susceptibility to Cryptosporidium infection, we used a modified model of weaned mice challenged with C. parvum oocysts and protein malnutrition simultaneously to study the mechanisms of their interaction. We found that C. parvum infection itself induced the expression of cleaved caspase 3 in intestinal epithelial cells, a well-known marker of cellular apoptosis, which had also been reported to be the key effector against parasite infection (16, 17). For the first time, we discovered that protein malnutrition, which worsens C. parvum infection, inhibited caspase-dependent apoptosis as well as epithelial cell proliferation, resulting in lower epithelial cell turnover and less cell shedding. This may allow C. parvum-infected cells to persist, underlying the interaction between C. parvum infection and protein malnutrition.

## MATERIALS AND METHODS

### Animal husbandry.

This study included the use of mice. This study was carried out in strict accordance with the recommendations in the *Guide for the Care and Use of Laboratory Animals* (18). The protocol was approved by the Committee on the Ethics of Animal Experiments of the University of Virginia (Protocol no. 3315). All efforts were made to minimize suffering. This protocol was approved and is in accordance with the Institutional Animal Care and Use Committee policies of the University of Virginia. The University of Virginia is accredited by the Association for the Assessment and Accreditation of Laboratory Animal Care (AAALAC).

The mice used in this study were male, 22 days old, of the C57BL/6 strain, and were ordered from Jackson Laboratories (Bar Harbor, ME). Mice weighed approximately 11 g on arrival and were cohoused in groups of up to five animals per cage. The vivarium was kept at a temperature of between 68 and 74°F with a 14-h light and 10-h dark cycle.

### Rodent diet.

Weaned mice (22 days old) were acclimated, fed a regular diet for 7 days, and then fed a protein source-defined control diet (20% protein [dN]) or protein-deficient diet (2% protein [dPD]) (Research Diets, Inc.). All diets were isocaloric, and calories from fat, protein, and carbohydrates are shown in Table S1 in the supplemental material. The amounts of each diet consumed were not significantly different between dN and dPD as determined in previous experiments (data not shown).

### Cryptosporidium parvum infection.

Oocysts of C. parvum (Iowa isolate) were purchased from Bunch Grass Farms (Deary, ID). The concentration of the stock solution, as received from the vendor (1 × 10^9^/50 ml phosphate-buffered saline [PBS]) was measured using a hemocytometer to estimate the number of oocysts needed. Each infected mouse received an inoculum of 2 × 10^7^ C. parvum unexcysted oocysts in 100 μl of freshly prepared oocyst solution via oral gavage directly into the stomach; controls received 100 μl PBS alone.

In this study, we used the following four groups: nourished uninfected (*n* = 3), malnourished uninfected (*n* = 3), nourished infected (*n* = 3), and malnourished infected (*n* = 3). At postnatal day 28, mice assigned to the nourished groups received 20% control dN diet for 20 h, whereas mice assigned to the malnourished groups received the isocaloric diet containing 2% protein (Research Diets) for 20 or 72 h. The specific diet and C. parvum infection were given at the same time. All experiments were repeated at least 3 times.

### Isolation of epithelial cells and protein extraction.

After rapid dissection of the mouse intestines, intestinal sections were cut longitudinally and rinsed in Hanks' balanced salt solution (HBSS) to remove luminal contents. Then intestinal tissues were placed in HBSS–50 mM EDTA–1 mM dithiothreitol (DTT) solution with shaking at 250 rpm at 37°C for 30 min. Next, the above solutions were poured through a cell strainer (100-μm pores) into 50-ml conical tubes, followed by centrifugation at 1,000 rpm at 4°C for 10 min. After discarding supernatant, cells were lysed in radioimmunoprecipitation assay (RIPA) buffer (20 mM Tris [pH 7.5], 150 mM NaCl, 1% Nonidet P-40, 0.5% sodium deoxycholate, 1 mM EDTA, 0.1% SDS) containing protease inhibitor cocktail (Roche) and phosphatase inhibitors (1 mM sodium orthovanadate, 5 mM sodium fluoride, 1 mM microcystin LR, and 5 mM β-glycerophosphate). Tissue lysates were cleared by centrifugation, and the supernatant was saved frozen at −80°C until use for Western blot analysis.

### Antibodies.

GAPDH (glyceraldehyde-3-phosphate dehydrogenase) antibody was purchased from Santa Cruz. Phospho-Akt (Ser473) antibody was purchased from Thermo Fisher Scientific, Inc. All other antibodies were obtained from Cell Signaling Technology.

### Western blotting.

Protein extracts were mixed with 4× Laemmli sample buffer, boiled for 5 min, loaded onto an SDS gel, and then transferred to a nitrocellulose membrane for Western blotting. After being blocked for 1 h in 5% dry milk, the membrane was incubated with primary antibody (1:1,000) in Tris-buffered saline (TBS) containing 0.1% Tween 20 and 5% bovine serum albumin either for 1 h at room temperature or overnight at 4°C, followed by extensive rinses and a 1-h incubation with fluorescence-conjugated secondary antibody (1:5,000). Fluorescence signals were detected with a Typhoon Trio+ variable mode imager and analyzed with the ImageJ software.

### DNA extraction.

DNA was isolated from fecal pellets using the QIAamp DNA stool minikit as previously described. DNA from tissue samples was extracted from frozen tissue samples using the QIAamp DNA tissue kit (Qiagen). To enhance the pathogen's DNA extraction, we made an improvement in the original protocol: a vigorous homogenization of the samples with 300 mg of 1.0-mm zirconia beads (BioSpec, Bartlesville, OK) using a MiniBeadBeater (BioSpec, Bartlesville, OK). DNA from cecum content or stool was extracted from the thawed stool samples using the QIAamp DNA stool kit (Qiagen) following the manufacturer's instructions. After extraction, DNA was eluted in 200 μl elution buffer and stored at −20°C.

### Real-time PCR for C. parvum quantification.

Stool and tissue DNA were analyzed for the C. parvum-specific CP15 gene to determine shedding of organisms in stool or tissue burden (19). Additionally, stool DNA was probed for murine β-actin to measure the amount of host-shed cells in the feces.

Quantification of the infection was performed in a Bio-Rad CFX PCR detection system by interpolating threshold cycle (*C_T_*) values of each run with a standard curve of known amounts of C. parvum DNA and transformed into the number of organisms per milligram of tissue sample. The master mix solution and primers were used as described elsewhere (3). Amplification consisted of 15 min at 95°C, followed by 40 cycles of 15 s at 95°C, 15 s at 52°C, and 20 s at 72°C and then 40 cycles of 10 s, starting at 75°C, with 0.5°C increments for the melt curve.

### Intestinal morphometry.

Ileal segments were fixed in 4% paraformaldehyde, embedded in paraffin, and stained with hematoxylin-eosin at the University of Virginia Histology Core.

### Immunohistochemistry.

Ileal samples were sectioned at 5 μm, placed in 10 mM citrate buffer of pH 6.0, and heated for 10 min. Sections were incubated for 10 min in 3% hydrogen peroxide (Sigma-Aldrich), washed with distilled water and phosphate-buffered saline for 5 min each, blocked in 10% normal horse serum in PBS for 1 h at room temperature (RT), and then incubated with one of the primary antibodies overnight at 4°C: cleaved caspase-3 (Asp175) monoclonal antibody or Ki67 monoclonal antibody (Cell Signaling, Boston, MA). After being washed, sections were incubated with conjugated horse anti-rabbit IgG (Vector Laboratories, Burlingame, CA) for 1 h at RT, washed, and incubated for1 h at RT with R.T.U. Vectastain Elite ABC reagent, according to the manufacturer's protocol (Vector Laboratories). After the washings, the section was developed with a 3,3′-diaminobenzidine (DAB) substrate kit (Vector Laboratories), to give a brown color. Slides were dehydrated in serial ethanol and xylene solution and permanently mounted. Images were digitally captured at ×200 using an Olympus BX51 microscope, and quantification of cleaved caspase-3 and Ki67 staining was performed in a blind manner by counting positive cells in multiple random microscope fields per tissue section.

### Statistical analysis.

Data analyses were performed with GraphPad Prism 5 software (GraphPad Software). All statistical analyses were done from raw data with analysis of variance, Student *t* tests, and Bonferroni *post hoc* analysis where applicable. Differences were considered significant at a *P* value of <0.05. Data are represented as means ± standard errors of the mean. All experiments were repeated a minimum of three separate times.

## RESULTS

### Protein malnutrition intensified C. parvum infection in a murine model. (i) Stool shedding of parasites.

**FIG 1:**
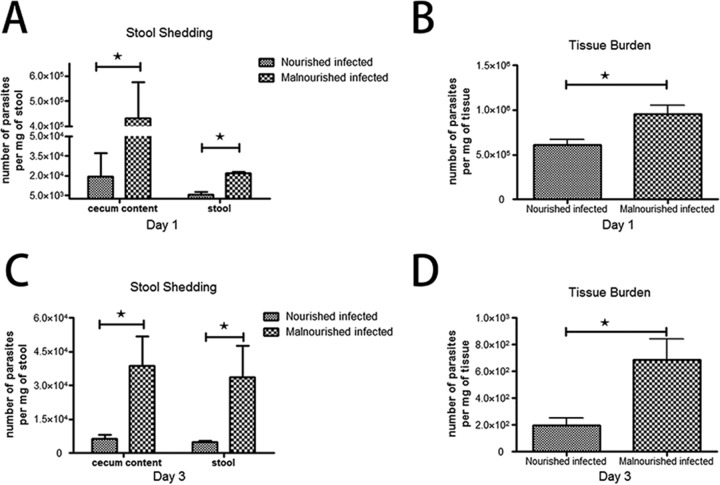
Tissue burden of C. parvum organisms in the ileum and stool shedding of challenged mice, as altered by nutritional status. C57BL/6 mice, nourished or malnourished, were challenged with 2 × 10^7^ unexcysted oocysts per mouse and then euthanized 20 or 72 h later in order to determine the level of ileal tissue burden of organisms and parasite shedding. The number of parasites per milligram of tissue or stool was determined by quantitative PCR. Data are shown as the mean ± standard error of the mean (SEM) (*n* = 3 mice per group). *, *P* < 0.05 for malnourished infected versus nourished infected.

To test the intensity of enteric infection in our new simultaneous challenge model, we quantified the number of C. parvum organisms shed into stool. Our result suggested that both malnourished and nourished mice were successfully infected, but the intensity of infection, as mirrored by shedding of organisms into the stool, was markedly increased in malnourished mice at both 20 and 72 h after infection (Fig. 1A and C).

### (ii) Tissue burden of organisms.

Distal ileums were taken from C57BL/6 mice challenged with C. parvum at different states of nutrition to test the effect of the organism on tissue burden. Malnourished mice showed significantly increased levels of intestine-associated C. parvum organisms compared with nourished mice, reflecting the intensified ability of the organism to infect the epithelium (Fig. 1B and D).

### (iii) Histopathologic changes.

**FIG 2:**
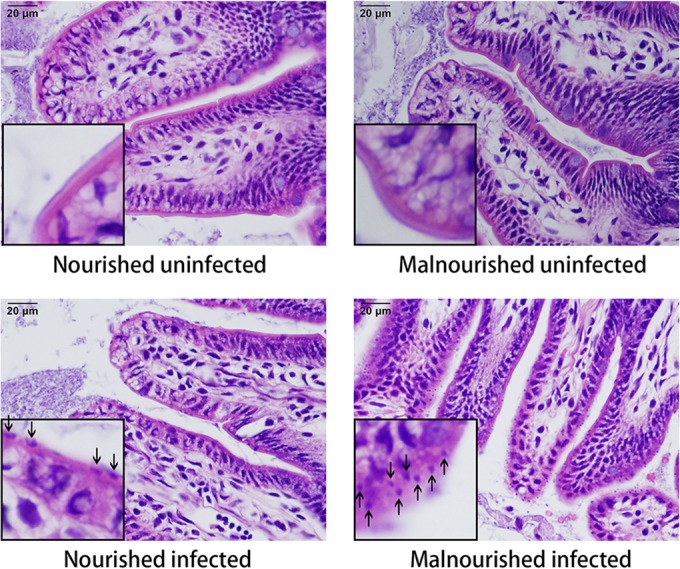
Ileal histology in nourished and malnourished uninfected and infected mice. Images in the left lower corner of each picture represent high-power magnification of a selected villus, showing cryptosporidial parasites at the enterocyte apical surface of infected mice (arrows). Hematoxylin and eosin, ×400.

The different groups did not show significant differences in villous height or crypt depth at that early stage of challenge. However, more C. parvum organisms were found at the enterocyte apical surface of malnourished infected mice compared with nourished infected ones. Moreover, C. parvum parasites were located mostly in the tips and sides of villus but not in crypts. Representative ileal histopathology from infected and uninfected nourished and malnourished mice is shown in Fig. 2 (20 h after challenge) and Fig. S1 in the supplemental material (72 h after challenge).

### Protein deprivation and C. parvum challenge influenced weight gain.

**FIG 3:**
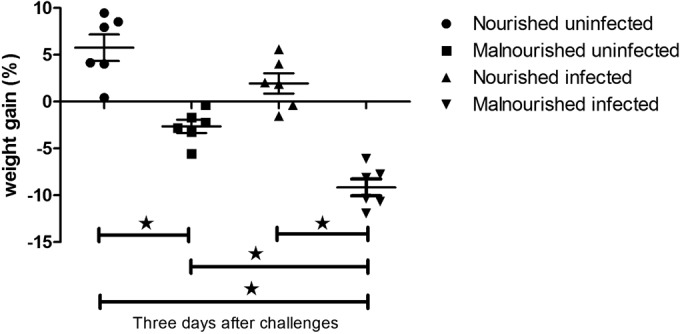
Growth of mice as altered by nutritional status and C. parvum infection. C57BL/6 mice at postnatal day 28 were fed with an isocaloric low-protein (2% protein) diet or control diet containing 20% protein and simultaneously challenged with 2 × 10^7^ unexcysted oocysts per mouse. Shown is the body weight change from the start of challenges until 3 days (72 h) after challenges. *, *P* < 0.05.

To show growth affected by a protein deficiency diet and C. parvum challenge, we measured the body weight change from the start of challenges until the endpoint of this experiment (72 h after challenges). The nourished uninfected mice gained weight as expected; C. parvum infection did not affect their growth, as shown in body weight change. Malnourished mice lost weight even without infection, but challenge with C. parvum significantly increased the weight loss (Fig. 3).

### Protein malnutrition impaired the increase of cleaved caspase 3 induced by C. parvum infection.

**FIG 4:**
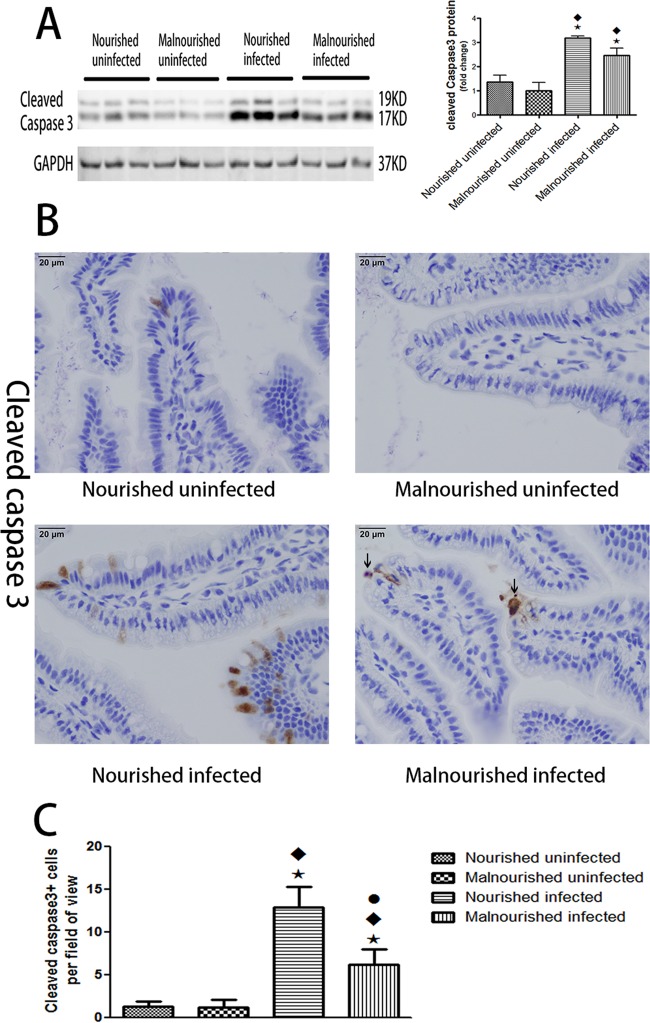
Protein malnutrition attenuated the expression of cleaved caspase 3 induced by C. parvum. C57BL/6 mice at postnatal day 28 were fed with an isocaloric low-protein (2% protein) diet or control diet containing 20% protein and simultaneously challenged with 2 × 10^7^ unexcysted oocysts per mouse and then euthanized 20 h later. (A) Equal amounts of total proteins from ileal epithelial cells were Western blotted against cleaved caspase 3 and GAPDH antibodies. After densitometry quantification and normalization against GAPDH, the fold change of the protein level relative to the nourished uninfected group is shown as the mean ± SEM (*n* = 3). (B) Immunostain of uninfected or infected ileum with different nutritional statuses for cleaved caspase 3. Arrows in the right lower image show shedding cells under apoptosis. (C) Quantification of cleaved caspase 3-positive epithelial cells in ileum. Cleaved caspase 3-positive cells were counted in five fields for each mouse. *, *P* < 0.05 compared with nourished uninfected; ⬥, *P* < 0.05 compared with malnourished uninfected; ●, *P* < 0.05 compared with nourished infected. Similar results were obtained from three independent experiments.

Since apoptosis has been shown to have a critical role in parasite infection, including cryptosporidiosis, we tried to investigate the expression of cleaved caspase 3, which is a well-known marker of apoptosis in ileal epithelial cells from the above model (20, 21). Western blot results showed an increase in expression of cleaved caspase 3 induced by C. parvum infection at just 20 h after infection. Meanwhile, protein malnutrition itself slightly decreased the expression of cleaved caspase 3 in uninfected mice and also attenuated the increase of cleaved caspase 3 induced by C. parvum infection (Fig. 4A). Similar patterns of expression of cleaved caspase 3 were found 72 h after challenge (see Fig. S2 in the supplemental material). Immunostaining showed that cleaved caspase 3-positive cells were mostly located at villus tips: there were significantly more cells in the nourished infected group than in the malnourished infected group (Fig. 4B and C).

### Protein malnutrition suppressed intestinal epithelial proliferation induced by C. parvum.

**FIG 5:**
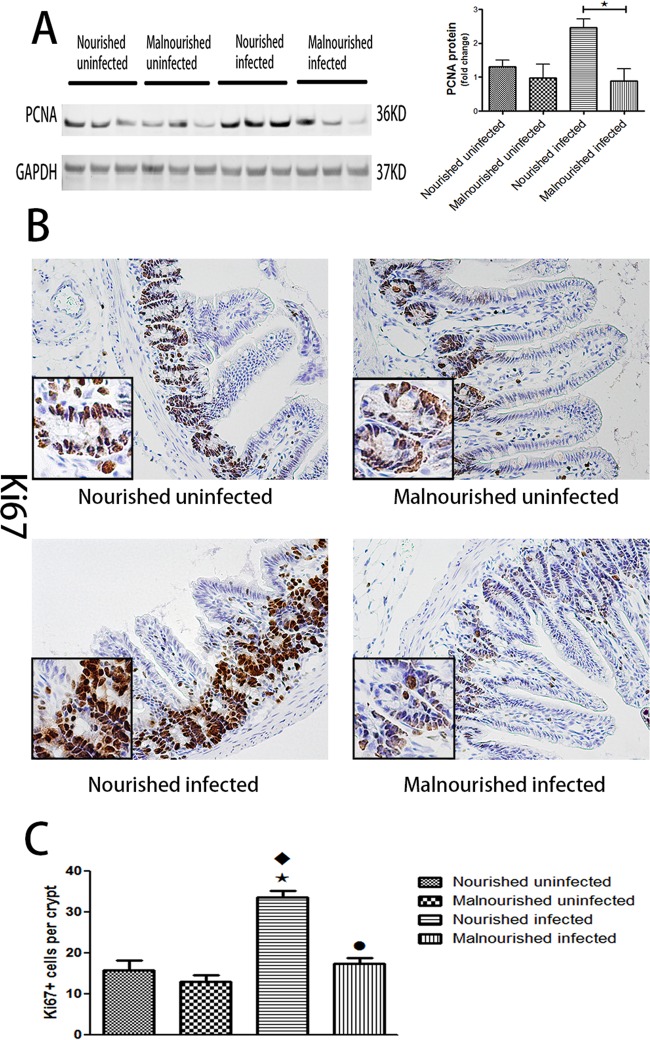
Protein malnutrition suppressed intestinal epithelial cell proliferation induced by C. parvum. C57BL/6 mice at postnatal day 28 were fed with an isocaloric low-protein (2% protein) diet or control diet containing 20% protein and simultaneously challenged with 2 × 10^7^ unexcysted oocysts per mouse and then euthanized 20 h later. (A) Equal amounts of total proteins from ileal epithelial cells were Western blotted against PCNA and GAPDH antibodies. After densitometry quantification and normalization against GAPDH, the fold change of the protein level relative to nourished uninfected group is shown as the mean ± SEM (*n* = 3). (B) Immunostain of uninfected or infected ileum with different nutritional statuses for Ki67. (C) Quantification of Ki67-positive epithelial cells in ileal crypts. Ki67-positive cells were measured in 10 crypts each mouse (*n* = 3). *, *P* < 0.05 compared with nourished uninfected; ♦, *P* < 0.05 compared with malnourished uninfected; ●, *P* < 0.05 compared with nourished infected. Similar results were obtained from three independent experiments.

Proliferation is the other part of intestinal epithelial homeostasis besides apoptosis. Our Western blot data showed an increase in expression of PCNA, a marker of cellular proliferation caused by C. parvum infection in the nourished infected group, while protein malnutrition suppressed the expression of PCNA at 20 and 72 h postinfection (Fig. 5A; see Fig. S2 in the supplemental material). Immunostaining of another proliferative marker, Ki67, confirmed the enhanced effect of C. parvum and the suppressed effect of protein malnutrition on proliferation of intestinal epithelial cells (Fig. 5B and C).

### Protein malnutrition decreased epithelial cell shedding induced by C. parvum infection.

**FIG 6:**
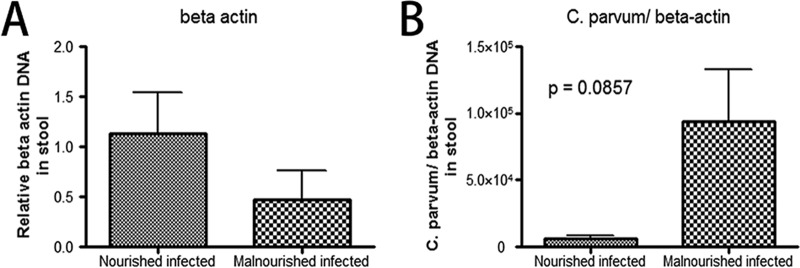
Stool DNA was probed for murine β-actin to measure the amount of host-shed cells in the feces. C57BL/6 mice, nourished or malnourished, were challenged with 2 × 10^7^ unexcysted oocysts per mouse and then euthanized 20 h later. (A) Stools were collected and DNA extracted for measurement of murine β-actin. The fold change of the DNA level relative to nourished infected group is shown as the mean ± SEM (*n* = 3). (B) Numbers of C. parvum organisms in stool were normalized against the DNA level of murine β-actin.

Primers for murine-specific β-actin were used to measure the amount of β-actin in stool from nourished or malnourished mice challenged with unexcysted oocysts of C. parvum (22). Since feces disable the investigation of shedding cells inside intestinal lumen, the quantity of murine-specific β-actin is a better way to reflect host-shed cells. Our results showed protein malnutrition decreased cell shedding, while each cell contained more parasites (Fig. 6).

### Protein malnutrition activated the Akt pathway.

**FIG 7:**
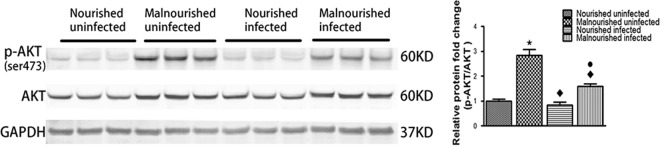
Protein malnutrition activated the Akt cell signaling pathway. Equal amounts of total proteins from ileum were Western blotted against total Akt, phosphorylated Akt (Ser473), and GAPDH antibodies. *, *P* < 0.05 compared with nourished uninfected; ⬥, *P* < 0.05 compared with malnourished uninfected; ●, *P* < 0.05 compared with nourished infected.

In order to find out mechanisms responsible for suppression of caspase 3 activity, we assessed the status of the Akt signaling pathway, which has been reported to inhibit caspase 3 activity and apoptosis in murine intestine (23, 24). Associated with decreased expression of cleaved caspase 3, the protein-deficient diet activated the Akt pathway by increasing the total Akt protein level and its Ser473 phosphorylation (Fig. 7).

## DISCUSSION

Much of the research concerning cryptosporidiosis has been done *in vitro* (20, 25, 26). Animal models for exploring C. parvum infection *in vivo* are limited, the majority of which use neonatal or immunosuppressive hosts (27–29). We have previously reported a model in the weaned malnourished mouse that more closely mimics the complex interaction between the normal immune response and pathogen (13, 14). We modeled the effects of malnutrition and cryptosporidiosis on growth rate and stool shedding, also confirming a vicious cycle by which malnutrition itself can increase vulnerability to C. parvum infection, while the intensified infection in turn leads to further malnutrition. However, we did not examine the early stage after infection. As changes in major signaling pathways that govern intestinal cellular response to stress were found in our previous work to occur mostly at 24 h after challenge, we modified our model with weaned mice and gave them the double challenge of a protein-deficient diet and C. parvum infection at the same time, and then examined the early changes at days 1 and 3 postinfection (15). We successfully reproduced the intensified C. parvum infection with protein deficiency, as shown by increased stool shedding of parasites and increased intestine-associated organisms. Moreover, weight loss was more serious in the group that was protein deficient and infected. Compared with the neonatal mouse model and the piglet model, our young adult mouse model has the advantages of being easily executed and less expensive (28, 30).

Caspases are cysteinyl aspartate-specific proteases that play a critical role in the induction of cellular apoptosis (21). Among members of the caspase family, activation of caspase 3 is considered to be a point at which a cell marches toward irreversible apoptotic death (16, 17). It has been reported that the host will limit the spread of infection by eliminating infected cells through activation of caspases and induction of apoptosis (21). *In vitro* studies have showed the essential role of apoptosis for the pathogenesis of C. parvum. Paradoxically, both increasing apoptosis by silencing Bcl-2 and decreasing apoptosis by the pan-caspase inhibitor impaired C. parvum infection (20). Although whether apoptosis of intestinal epithelial cells benefits either the host or the pathogen still needs to be determined, caspase-dependent apoptosis was undoubtedly increased by C. parvum in both *in vitro* and *in vivo* models (26, 28, 30). In the present study, we showed the evidence in a murine model concerning the relationship between C. parvum infection and caspase-dependent apoptosis of epithelial cells. Moreover, a protein-deficient diet was discovered to inhibit caspase 3 activity of intestinal epithelial cells, which in turn intensified C. *parvum* infection. This observed delay in intestinal epithelial turnover may be the possible mechanism of the vicious cycle between protein malnutrition and C. parvum infection.

A single layer of epithelial cells makes up the barrier against antigens, toxins, and microorganisms inside the gastrointestinal lumen. These epithelial cells differentiate from stem cells in the crypt, migrate gradually up the villus, and are shed at the villus tip under the apoptotic state (31, 32). Caspase-dependent apoptosis has been shown to mediate epithelial cell death and shedding at the villus tip (30, 33). Moreover, the host was found to eliminate C. parvum by increasing epithelial apoptosis and shedding infected cells at the villus tip in a piglet model (30). Protein malnutrition decreased caspase-dependent apoptosis of epithelial cells in the present study, as well as slowing crypt cell proliferation. This slowdown in epithelial cell turnover may cause the host to retain the infected cells longer, thus enhancing C. parvum parasite replication in the epithelium. In order to find supportive evidence in cell shedding, we first tried to count the shedding cells in intestinal lumen. However, feces inside the lumen affected our investigation. Then we used a mouse-specific probe to detect genomic β-actin in the stool. Although not significant, there was less expression of β-actin in the protein malnutrition group. This indicated that there were fewer shedding cells in malnourished group, but each cell bore more C. parvum organisms, which was consistent with our hypothesis.

The ongoing process of not only epithelial apoptosis at villus tips, but also proliferation in crypts, mediates epithelial cell turnover, their coordination maintaining epithelial homeostasis (31, 34). Besides inducing epithelial apoptosis, an increase in epithelial cell proliferation was also found after C. parvum infection in the present model, which is consistent with previous reports in the literature (35, 36). Increasing both cell shedding and proliferation in intestinal epithelial cells indicated a defensive response of host to C. parvum infection—maintaining the gut barrier while eliminating infected cells. In contrast, protein malnutrition, which drives epithelial cells into a “survival mode,” slowed down the epithelial cell turnover rate and impaired this protective reaction. Parasites like C. parvum and Giardia lamblia adhere to intestinal epithelial cells during infection (5, 37). The impaired epithelial cell turnover may help these parasites to persist with the infected epithelium, a mechanism that may explain why protein malnutrition intensified the infection of these two parasites in our previous studies (13, 38). However, it is still uncertain whether the change in epithelial proliferation is just a feedback to the state of cellular apoptosis or is another part of initiative defense.

The phosphatidylinositol 3-kinase (PI3K)/Akt signaling pathway is a major cell survival pathway. Its antiapoptotic role in intestinal epithelial cells of murine models has been shown in many studies (23, 24, 39). Our previous study demonstrated that the small intestine responds to protein malnutrition by activating major survival pathways and entering a “survival mode” (15). Activation of Akt pathways was found in our previous study and the present study, which may play a role in increasing cellular resistance to apoptosis. This may be the upstream mechanism for mediating caspase 3 activity in our model. However, the mechanism of inhibited proliferation by protein malnutrition still needs further study.

Our study has some limitations. First, we do not have direct evidence to show intestinal epithelial cell shedding is suppressed by protein malnutrition. Second, means to enhance intestinal epithelial turnover have not been used to extend our hypothesis. Further studies are planned to extend these observations and to examine potential therapeutic interventions that may enhance epithelial turnover and attenuate the effect of protein malnutrition on infection with C. parvum and other parasites.

In summary, the present study has provided novel insights into mechanisms of interaction between protein malnutrition and cryptosporidiosis. We find that protein malnutrition rapidly activates PI3K/Akt signaling pathway, inhibits caspase-dependent apoptosis, impairs turnover of epithelial cells, and intensifies C. parvum infection in a murine model. These findings provide a potential mechanism for the vicious cycle of malnutrition and parasitic infection as well as a potential strategy to eliminate C. parvum by using approaches that enhance cell turnover and renewal to thus enable increased clearance of infected epithelial cells.

## Supplementary Material

Supplemental material
