# Supplementary material for: Protein Malnutrition Impairs Intestinal Epithelial Cell Turnover, a Potential Mechanism of Increased Cryptosporidiosis in a Murine Model
Source: Infect Immun. 2016 Nov 18;84(12):3542–9. doi: 10.1128/IAI.00705-16 (PMC5116730; doi:10.1128/IAI.00705-16)
Supplement: Supplemental material [file supp_84_12_3542__index.html]

Supplemental material 

# Protein Malnutrition Impairs Intestinal Epithelial Cell Turnover, a Potential Mechanism of Increased Cryptosporidiosis in a Murine Model

## Supplemental material

**Files in this Data Supplement:**

- Supplemental file 1 -

  Table S1. Rodent diet composition. Fig. S1. Ileal histology in nourished and malnourished uninfected and infected mice at 72 h after challenge. Fig. S2. Protein malnutrition suppressed the expression of cleaved caspase 3 and PCNA induced by *C. parvum* at 72 h after challenge.

  PDF, 251K
