# Supplementary material for: Protein Malnutrition Impairs Intestinal Epithelial Cell Turnover, a Potential Mechanism of Increased Cryptosporidiosis in a Murine Model
Source: Infect Immun. 2016 Nov 18;84(12):3542–9. doi: 10.1128/IAI.00705-16 (PMC5116730; doi:10.1128/IAI.00705-16)
Supplement: Supplemental material [file IAI.00705-16_zii012161911so1.pdf]

Supplemental Table 1. Rodent diet composition

	dN		dPD	
%	gm	kcal	gm	kcal
Protein	20	20.5	2	2
Carbohydrate	66.3	68	84	86
Fat	5	11.5	5	12
Total		100		100
kcal/gm	3.9		3.9	
**Ingredient**	**gm**	**kcal**	**gm**	**kcal**
Egg Whites, Spray Dried	200	800	20	80
Corn Starch	150	600	330	1320
Sucrose	502.5	2010.2	502.5	2010
Cellulose, BW200	50	0	50	0
Corn Oil	50	450	50	450
Mineral Mix S19401	35	0	35	0
Vitamin Mix V10001	10	40	10	40
Choline Bitartrate	2	0	2	0
Biotin, 1%	0.4	0	0.4	0
Zinc Carbonate, 52.1% Zinc	0.056	0	0.056	0
**Total**	**1000**	**3900**	**1000**	**3900**

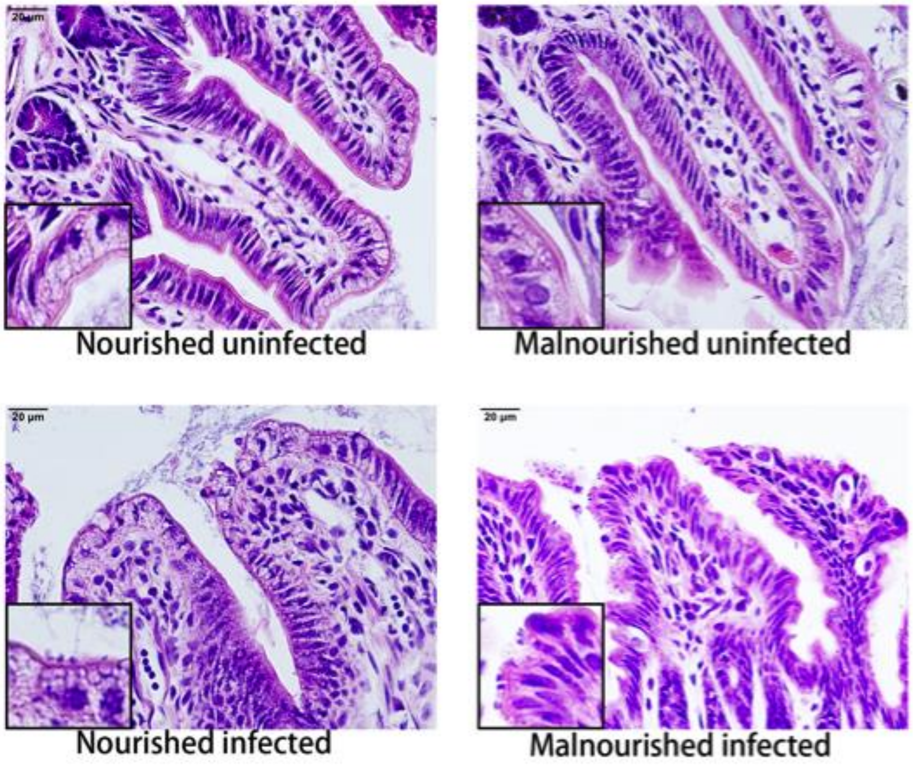

FIG S1 Ileal histology in nourished and malnourished uninfected and infected mice at 72 hours after challenge. Images in left-lower corner of each picture represented high-power magnification of a selected villus, showing cryptosporidial parasites at the enterocyte apical surface of infected mice, H&E ×400.

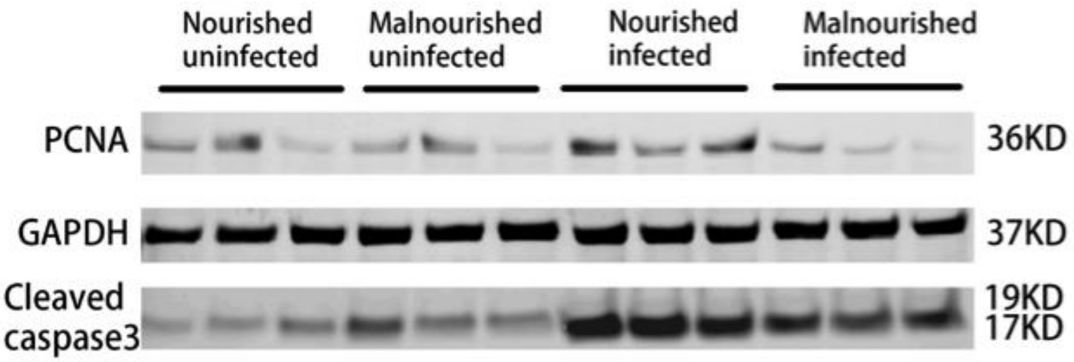

FIG S2 Protein malnutrition suppressed the expression of cleaved caspase 3 and PCNA induced by *C. parvum* at 72 hours after challenge. C57BL/6 mice at postnatal day 28 were fed with an isocaloric low-protein (2% protein) diet or the regular diet containing 20% protein and simultaneously challenged with 2×10^7 unexcysted oocysts per mouse, then euthanized 72 hours later. Equal amount of total proteins from ileal epithelial cells were Western blotted against Cleaved caspase 3, PCNA and GAPDH antibodies.
